# Ginsenoside-Rb3 protects the myocardium from ischemia-reperfusion injury via the inhibition of apoptosis in rats

**DOI:** 10.3892/etm.2014.2007

**Published:** 2014-10-07

**Authors:** XIAOMIN LIU, YICHUAN JIANG, XIAOFENG YU, WENWEN FU, HONG ZHANG, DAYUN SUI

**Affiliations:** 1 Department of Pharmacology, School of Pharmaceutical Sciences, Jilin University, Changchun, Jilin 130021, P.R. China; 2 Department of Pharmacy, Affiliated Hospital of Jilin Medical College, Jilin, Jilin 132013, P.R. China

**Keywords:** ginsenoside-Rb3, myocardium, ischemia-reperfusion injury, apoptosis

## Abstract

Ginsenoside-Rb3 (G-Rb3) has been previously demonstrated to attenuate myocardial ischemia-reperfusion injury (MIRI). The aim of the present study was to investigate this further and determine whether G-Rb3 protects the myocardium from ischemia-reperfusion injury via the inhibition of apoptosis. Adult male Sprague Dawley rats were randomly divided into four groups: Sham, MIRI, G-Rb3 treatment (orally, 20 mg/kg) and ischemic postconditioning (as the positive control). The drug or placebo treatment was administered to the rats once a day for three consecutive days, and MIRI was then induced by subjecting the rats to left anterior descending coronary artery ligation for 30 min and reperfusion for 2 h. The results showed that G-Rb3 treatment significantly reduced the number of apoptotic cells in the myocardium and the expression of B-cell lymphoma 2-associated X protein, and increased the expression of B-cell lymphoma 2. The activities of aspartate aminotransferase, lactate dehydrogenase and creatine kinase-MB in the serum were also reduced significantly by the G-Rb3 treatment. These findings suggest that G-Rb3 inhibits apoptosis in the early stage of MIRI, and attenuates MIRI when the reperfusion continues. G-Rb3 was also shown to significantly reduce the level of malondialdehyde and increase the activity of superoxide dismutase in the myocardium, which suggests that attenuating reactive oxygen species accumulation and oxidative stress may be the major mechanism underlying the anti-apoptotic effects of G-Rb3. The release of inflammatory factors was significantly attenuated by G-Rb3, which may also be associated with its anti-apoptotic effects.

## Introduction

Acute myocardial infarction (AMI) is one of the major diseases causing clinical mortality (1). In the past decade, doctors have been able to make the ischemic heart regain blood perfusion and restore oxygen supply in a short time using modern medical technologies, including thrombolytic therapy and percutaneous coronary vessel intervention; however, coronary reperfusion typically causes myocardial ischemia-reperfusion injury (MIRI), leading to conditions including arrhythmia, expansion of the infarct size and persistent ventricular systolic dysfunction. MIRI is the main reason for the poor prognosis of AMI (2,3).

Apoptosis is the process of programmed cell death that occurs in multicellular organisms. Biochemical events lead to characteristic cell changes and death. Previous studies have shown that cardiomyocyte apoptosis plays an important role in the processes of AMI and MIRI (4–6); therefore, the development of drugs that can attenuate MIRI, inhibit cardiomyocyte apoptosis and improve cardiac function has an important clinical significance.

The medicinal herb *Panax quinquefolium*, also known as American ginseng, has a long history of use in a number of countries, including China (7,8). Ginsenosides are the major active components of *P. quinquefolium* (7,8), which has been described to possess anti-stress, anti-diabetic and antitumor effects (9–12). Ginsenoside-Rb3 (G-Rb3), an extract from the stems and leaf of *P. quinquefolium*, has been demonstrated to exhibit a variety of protective effects in the cardiovascular system by our laboratory (13–15), including attenuating MIRI in rats (15).

In our previous study (15), Sprague Dawley rats were orally treated with G-Rb3 daily for three days, prior to being subjected to left anterior descending coronary artery ligation for 30 min and reperfusion for 24 h. The results showed that G-Rb3 treatment resulted in a reduction in myocardial infarct size, as well as in creatine kinase-MB (CK-MB) activity and lactate dehydrogenase (LDH) activity in the serum. The cardioprotective effect of G-Rb3 was further confirmed by histopathological examination. Whether G-Rb3 protects the myocardium from ischemia-reperfusion injury via inhibition of apoptosis, however, is yet to be elucidated. Previous studies (4–6) showed that MIRI and apoptosis appear in the reperfused myocardium within ≤4 h; therefore, reperfusion for 24 h is not the optimal time-period to observe the anti-apoptotic effects of drugs. In the present study, the time of reperfusion was reduced to 120 min to observe the functional and histopathological changes in the early stage of MIRI. The aim of this study was to verify the anti-apoptotic effects of G-Rb3 and analyze the mechanism underlying its cardioprotective effects. Ischemic postconditioning (IP), which has been proven to attenuate MIRI (16), was selected to be the positive control.

## Materials and methods

### Chemicals and reagents

A terminal deoxynucleotidyl-transferase-mediated dUTP nick end labeling (TUNEL) apoptosis detection kit was purchased from Roche Diagnostics (Basel, Switzerland). Antibodies against B-cell lymphoma 2 (Bcl-2) and Bcl-2-associated X protein (Bax) for immunohistochemistry (IHC) were purchased from Cell Signaling Technology, Inc. (Beverly, MA, USA). Malondialdehyde (MDA) and superoxide dismutase (SOD) assay kits were purchased from Nanjing Jiancheng Bioengineering Institute (Nanjing, China). Tumor necrosis factor-α (TNF-α) and interleukin-6 (IL-6) radioimmunoassay kits were purchased from Beijing Kangyuan Ruide Biotechnology Co., Ltd. (Beijing, China). The G-Rb3 was obtained from Dr Yanping Chen (Department of Natural Medicinal Chemistry, School of Chemistry, Jilin University, Changchun, China) and dissolved in double-distilled (dd)H_2_O for use. All other chemicals were analytical reagents.

### Animals

Male Sprague Dawley rats (weight, 200–220 g) were purchased from the Experimental Animal Center of Jilin University (Changchun, China). All rats were allowed free access to food and water. The experiments were performed in accordance with the Guide for the Care and Use of Laboratory Animals of Jilin University, and approved by the Ethics Committee of Jilin University.

The rats were randomly divided into four groups (10 rats in each group): i) Sham surgery (sham), ii) MIRI, iii) G-Rb3 and iv) IP. Rats in the G-Rb3 group were orally administered 20 mg/kg G-Rb3 (dissolved in ddH_2_O) and the other three groups were treated with ddH_2_O. The drug or ddH_2_O treatment was administered once a day for three consecutive days. Thirty minutes after the final treatment, the rats were anesthetized for surgery.

### Surgical procedures

In the MIRI and G-Rb3 groups, myocardial ischemia was induced by exposing the heart through a left thoracic incision and placing a 6/0 silk suture with a slipknot around the left anterior descending coronary artery. After 30 min ischemia, the slipknot was released and the myocardium reperfused for 2 h. In the IP group, the rats were treated three times with 30 sec reperfusion and 30 sec ligation before the 2 h reperfusion, while the sham group underwent left thoracotomy only.

After 2 h reperfusion, heart rate (HR) and blood pressure were measured, and then the blood and myocardium tissue samples were obtained. Six out of the 10 rat hearts in every group had the apex cordis removed for assay of the MDA levels and the SOD activity, while the remaining cardiac tissue samples were fixed in 4% buffered paraformaldehyde solution for histopathological examination. The other four rat hearts were prepared for infarct size measurement.

### Measurement of infarct size

To distinguish between the normal and infarcted myocardium, the hearts were cut into four horizontal slices and incubated with *p*-nitro-blue tetrazolium (NBT; 0.5 mg/ml, 10 min at 37°C). In the presence of intact dehydrogenase enzyme systems (normal myocardium), NBT forms a dark blue formazan; by contrast, areas of necrosis lack dehydrogenase activity and therefore do not stain (17). The normal and infarcted myocardium were weighed together and then separated by following the line of demarcation between the dark blue stained and unstained tissue. The infarcted myocardium was then weighed. The infarct size was calculated as infarcted myocardium mass divided by total myocardium mass, and expressed as a percentage.

### Histopathological examination: Histology, TUNEL and IHC

The myocardial tissue samples were fixed in 4% buffered paraformaldehyde solution, and then embedded in paraffin. Four-micrometer thick paraffin sections were stained with hematoxylin and eosin (HE). TUNEL staining with the paraffin sections was performed according to the kit manufacturer’s instructions (Roche Diagnostics). The sections were examined using light microscopy (Nikon E100; Nikon Corporation, Tokyo, Japan), and photomicrograph images were captured.

For the IHC, endogenous peroxidase activity was blocked with 3% methanol-H_2_O_2_. Nonspecific sites were treated with a blocking buffer (5% albumin and 10% specific serum in phosphate-buffered saline). Primary antibody (against Bcl-2 or Bax) were added to the sections in the blocking buffer and incubated overnight at 4°C. Subsequent to washing, polyclonal goat anti-rat antibody (Beijing Dingguo Changsheng Biotech Co., Ltd., Beijing, China) was added and washed, prior to development with avidin-biotin-streptavidin complex and diaminobenzidine chromogen. Photomicrograph images were then captured.

The Motic Images Advanced 3.2 image analysis system (Motic, Causeway Bay, Hong Kong) was used to analyze the photomicrographs of the TUNEL and IHC staining, and the results were expressed by grayscale value. Higher grayscale values correlated with lighter TUNEL or IHC staining, which indicated fewer apoptotic cells or lower protein (Bcl-2 or Bax) expression.

### Assay of MDA levels and SOD activity

The preparation of the myocardium tissue was as follows: 1 g apex cordis was homogenized in nine volumes of ice-cold saline and centrifuged (1,500 × g) at 4°C for 15 min. The supernatant was obtained (stored at −80°C) to analyze the MDA levels and the SOD activity using assay kits and a spectrophotometer (7202B; Unico Instrument Co., Ltd., Shanghai, China), in accordance with the kit manufacturer’s instructions (Nanjing Jiancheng Bioengineering Institute).

### Assay of the TNF-α and IL-6 levels and the activities of aspartate aminotransferase (AST), CK-MB and LDH

Blood samples were collected and left at room temperature for 2 h to allow complete clotting and then centrifuged (1,500 × g) at 4°C for 15 min. The serum was removed and stored at −80°C for radioimmunoassay and biochemical assay. The TNF-α and IL-6 levels were analyzed using radioimmunoassay kits purchased from Beijng Kangyuan Ruide Biotechnology Co., Ltd. The activities of AST, LDH and CK-MB were analyzed by the Clinical Laboratory in the First Hospital of Jilin Academy of Traditional Chinese Medicine (Changchun, China), using commercial kits by employing an automatic biochemical analyzer (Cobas-Fara; Roche Diagnostics, Basel, Switzerland).

### Statistical analysis

All results are presented as the mean ± standard deviation. The Student’s t-test was applied when two or more values were compared.

## Results

### Effects of G-Rb3 on heart rate and blood pressure

Compared with the sham surgery control, MIRI induced heart function impairment; this was demonstrated by the significantly reduced HR and systolic blood pressure (SBP) in the MIRI group (P<0.05). Compared with the MIRI group, the HRs of the G-Rb3 and IP groups were significantly reduced (P<0.05), but no significant differences were observed in the SBP and diastolic blood pressure (Table I).

### Effects of G-Rb3 on infarct size

**Figure 1:**
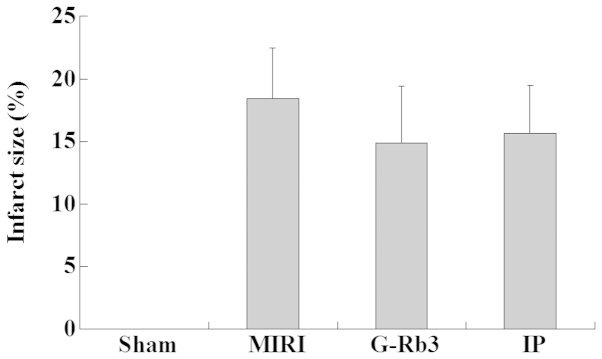
Effect of G-Rb3 on infarct size of the myocardium in MIRI rats. Data are presented as the mean ± standard deviation, n=4 for each group. MIRI, myocardial ischemia-reperfusion injury; G-Rb3, ginsenoside-Rb3; IP, ischemic postconditioning.

The infarct size of the myocardium in the sham group was zero, while the size in the MIRI group was 18.42±4.05%. Compared with the MIRI group, the infarct size was reduced in the G-Rb3 (14.85±4.58%) and IP (15.66±3.80%) groups, although the difference was not significant (Fig. 1).

### Effects of G-Rb3 on histology, TUNEL and IHC

**Figure 2:**
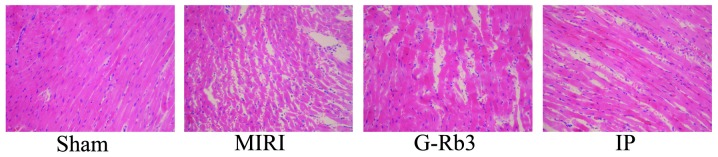
Representative hematoxylin and eosin staining histology photomicrographs of myocardium tissue. The sections were examined under a light microscope, prior to photomicrograph images being captured. MIRI, myocardial ischemia-reperfusion injury; G-Rb3, ginsenoside-Rb3; IP, ischemic postconditioning.

According to the HE staining, the myocardial tissue samples of the sham group exhibited no focal separation of myocardial fibers, but had a small amount of erythrocyte infiltration. The samples of the other three groups exhibited focal destruction of myocardial fibers with erythrocyte and neutrophil infiltration; however, myocyte necrosis was rarely observed. The condition of myocardial injury of these three groups was similar. (Fig. 2).

**Figure 3:**
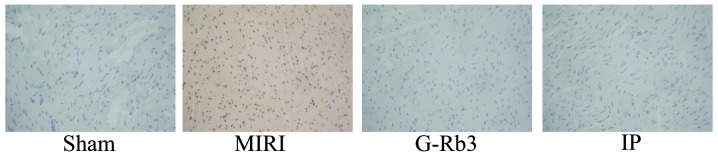
Representative terminal deoxynucleotidyl-transferase-mediated dUTP nick end labeling staining photomicrographs of myocardial tissue. The sections were examined under a light microscope, prior to photomicrograph images being captured. MIRI, myocardial ischemia-reperfusion injury; G-Rb3, ginsenoside-Rb3; IP, ischemic postconditioning.

**Figure 4:**
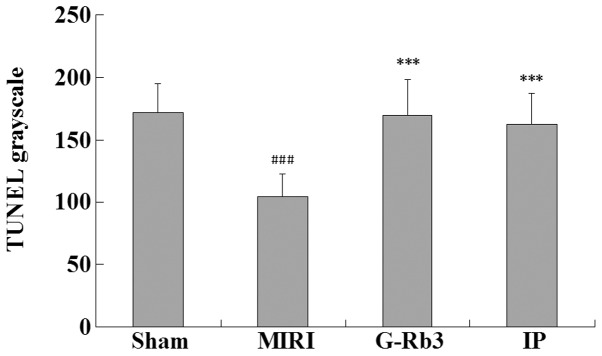
Effect of G-Rb3 on the grayscale of TUNEL staining photomicrographs. Data are presented as the mean ± standard deviation, n=6 for each group. ^###^P<0.001 compared with the sham group; ^***^ P<0.001 compared with the MIRI group. MIRI, myocardial ischemia-reperfusion injury; G-Rb3, ginsenoside-Rb3; IP, ischemic postconditioning; TUNEL, terminal deoxynucleotidyl-transferase-mediated dUTP nick end labeling.

**Figure 5:**
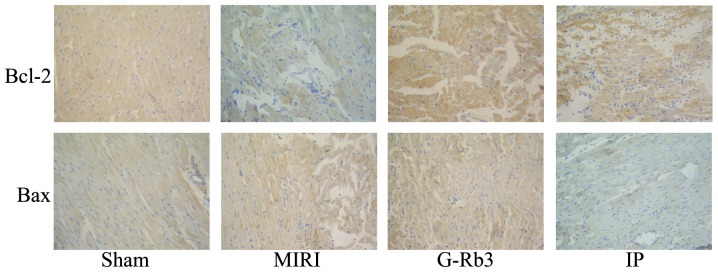
Representative immunohistochemistry staining photomicrographs of myocardial tissue. Antibodies against Bcl-2 and Bax were used as the primary antibodies. The sections were examined under a light microscope, prior to photomicrograph images being captured. MIRI, myocardial ischemia-reperfusion injury; G-Rb3, ginsenoside-Rb3; IP, ischemic postconditioning; Bcl-2, B-cell lymphoma 2; Bax, Bcl-2-associated X protein.

**Figure 6:**
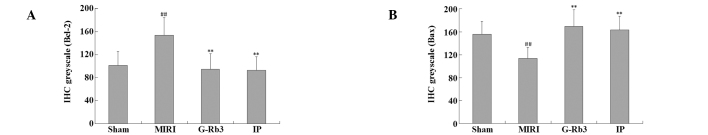
Effect of G-Rb3 on the grayscale of IHC staining photomicrographs. Antibodies against (A) Bcl-2 and (B) Bax were used as the primary antibodies. Data are presented as the mean ± standard deviation, n=6 for each group. ^##^P<0.01 compared with the sham group; ^**^P<0.01 compared with the MIRI group. MIRI, myocardial ischemia-reperfusion injury; G-Rb3, ginsenoside-Rb3; IP, ischemic postconditioning; Bcl-2, B-cell lymphoma 2; Bax, Bcl-2-associated X protein; IHC, immunohistochemistry.

Compared with the sham group, the numbers of apoptotic cells were increased significantly in the MIRI group and reduced significantly in the G-Rb3 and IP groups (P<0.001). (Figs. 3 and 4) The expression of Bcl-2 in the myocardial tissue samples was lower in the MIRI group than that in the other three groups, and this difference was significant (P<0.01). By contrast, the expression of Bax was higher in the MIRI group than that in the other three groups; these differences also showed significance (P<0.01) (Figs. 5 and 6).

### Effects of G-Rb3 on MDA levels and SOD activity

**Figure 7:**
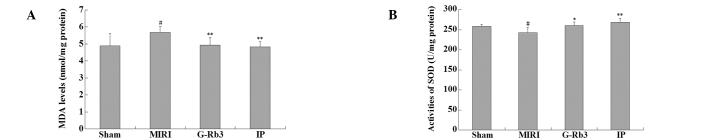
Effect of G-Rb3 on (A) MDA level and (B) the activity of SOD in myocardial tissue. Data are presented as the mean ± standard deviation, n=6 for each group. ^#^P<0.05 compared with the sham group; ^*^P<0.05 and ^**^P<0.01 compared with the MIRI group. MIRI, myocardial ischemia-reperfusion injury; G-Rb3, ginsenoside-Rb3; IP, ischemic postconditioning; MDA, malondialdehyde; SOD, superoxide dismutase.

The MDA levels in the myocardial tissue of the MIRI group were significantly higher than those of the sham group (P<0.05), while the SOD activity was significantly lower in the MIRI group than that in the sham group (P<0.05). G-Rb3 treatment significantly reduced the MDA levels (P<0.01) and improved the SOD activity (P<0.05); this was a similar trend to the IP group (P<0.01, MDA and SOD) (Fig. 7).

### Effects of G-Rb3 on TNF-α and IL-6 levels

**Figure 8:**
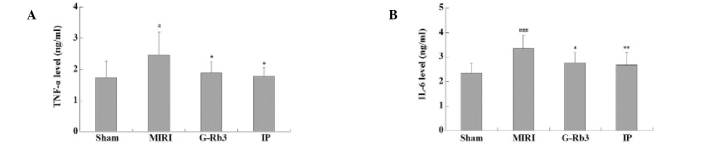
Effects of G-Rb3 on (A) TNF-α and (B) IL-6 levels in serum. Data are presented as the mean ± standard deviation, n=10 for each group. ^#^P<0.05 and ^###^P<0.001 compared with the sham group; ^*^P<0.05 and ^**^P<0.01 compared with the MIRI group. MIRI, myocardial ischemia-reperfusion injury; G-Rb3, ginsenoside-Rb3; IP, ischemic postconditioning; TNF-α, tumor necrosis factor-α; IL-6, interleukin-6.

The serum TNF-α levels of the MIRI group were significantly higher than those of the sham group (P<0.05), and the TNF-α levels of the G-Rb3 (P<0.05) and IP (P<0.05) groups were significantly reduced compared with those of the MIRI group. The findings were similar for IL-6 levels, which were significantly increased in the MIRI group (P<0.001) and significantly reduced in the G-Rb3 (P<0.05) and IP (P<0.01) groups (Fig. 8).

### Effects of G-Rb3 on the activities of AST, LDH and CK-MB

The activities of AST, LDH and CK-MB in the serum were significantly increased in the MIRI group (P<0.01) compared with those in the sham group. The G-Rb3 and IP treatments reduced the activities of AST, LDH and CK-MB, and the difference was significant (P<0.05) (Table II).

## Discussion

Apoptosis has been studied for decades. In mammals, there are two major pathways of apoptosis *in vivo*: The death receptor pathway and the mitochondrial death pathway (18). MIRI results in the accumulation of reactive oxygen species (ROS), which causes oxidative stress and mitochondrial injury (19,20). With the development of MIRI, the mitochondrial death pathway becomes activated, causing cardiomyocyte apoptosis and irreversible myocardial injury. In addition to ROS accumulation and oxidative stress, MIRI may also cause the release of inflammatory factors (21,22), particularly TNF-α, which is associated with the death receptor pathway.

As described in the Introduction, G-Rb3 treatment can attenuate MIRI in the rat heart with 30 min ischemia and 24 h reperfusion. The infarct size was reduced significantly and there was a significant difference in the histological result (15). In the present study, however, when the reperfusion time was reduced to 2 h, the differences in the infarct size and histological results between the MIRI and G-Rb3 groups were no longer significant. A possible reason is that in the early stage of MIRI, myocardial injury is reversible and the organic change is slight. During this stage, drug or IP/ischemic preconditioning treatment can inhibit cardiomyocyte apoptosis and attenuate MIRI by protecting the mitochondria from oxidative stress and reducing inflammation. When the reperfusion is continued, MIRI without treatment can therefore cause serious conditions, including arising arrhythmia, expansion of the infarct size and persistent ventricular systolic dysfunction. These serious conditions can be prevented with treatment, as shown in our previous study (15).

According to the histopathological examination, although the MIRI and G-Rb3 groups exhibited similar conditions in the photomicrographs of HE staining, the TUNEL results show that cardiomyocyte apoptosis was inhibited in the G-Rb3 group compared with the MIRI group. This was further verified by the IHC results, which showed that the expression of Bcl-2, one of the most important anti-apoptotic proteins (23), was increased with G-Rb3 treatment, and the expression of Bax, which has the opposite effect to Bcl-2, was reduced with G-Rb3 treatment. The results of the assays for MDA levels and SOD activity showed that G-Rb3 treatment attenuated ROS accumulation and oxidative stress, which may have been a major mechanism underlying its anti-apoptotic effect. The serum TNF-α and IL-6 levels were also reduced with G-Rb3 treatment, which may also have been associated with its anti-apoptotic effect. Further study is required to determine these mechanisms, particularly at the molecular level, and apoptosis-related genes and proteins, such as the caspase family, require analysis (24). The cardioprotective effect of G-Rb3 was confirmed by the AST, LDH and CK-MB results. Although G-Rb3 treatment did not improve the SBP reduced by MIRI, it did reduce the HR further, thus reducing the myocardial oxygen consumption and protecting the heart. According to the majority of the results, the cardioprotective effects of G-Rb3 are similar to those of IP.

In conclusion, G-Rb3 treatment can inhibit cardiomyocyte apoptosis in the early stage of MIRI in rats, consequently preventing serious conditions occurring when the reperfusion is continued. This is one of the major mechanisms of its cardioprotective effects, which is similar to IP. Attenuating ROS accumulation and oxidative stress may be a possible mechanism underlying the anti-apoptotic effect of G-Rb3, which is also associated with the reduction of inflammatory factor levels. Further studies at the molecular level to determine these mechanisms are a research direction of the future.

## Figures and Tables

**Table I:** Effects of G-Rb3 on heart rate and blood pressure.

Group	Heart rate (beats/min)	Systolic blood pressure (mmHg)	Diastolic blood pressure (mmHg)
Sham	477.1±36.1	154.4±22.4	104.4±34.4
MIRI	436.9±22.1^a^	120.0±9.3^a^	82.5±11.6
G-Rb3	406.0±34.9^b^	116.3±16.9	82.5±19.8
IP	402.4±35.5^b^	125.0±16.0	80.6±20.4

Data are presented as the mean ± standard deviation. N=10 for each group.

a P<0.05 compared with the sham group;

b P<0.05 compared with the MIRI group.

MIRI, myocardial ischemia-reperfusion injury; G-Rb3, ginsenoside-Rb3; IP, ischemic postconditioning.

**Table II:** Effects of G-Rb3 on AST, LDH and CK-MB.

Group	AST (U/ml)	LDH (U/ml)	CK-MB (U/ml)
Sham	402.1±208.8	1246.7±744.0	2308.3±945.0
MIRI	820.1±198.2^a^	2256.1±564.2^a^	3799.3±487.2^a^
G-Rb3	598.3±209.9^b^	1510.3±724.3^b^	3072.0±850.1^b^
IP	600.4±192.1^b^	1727.0±315.3^b^	3220.8±558.2^b^

Data are presented as the mean ± standard deviation, n=10 for each group.

a P<0.01 compared with the sham group;

b P<0.05 compared with the MIRI group.

MIRI, myocardial ischemia-reperfusion injury; G-Rb3, ginsenoside-Rb3; IP, ischemic postconditioning; AST, aspartate aminotransferase; LDH, lactate dehydrogenase; CK-MB, creatine-MB.

## References

[1] Chambless L, Keil U, Dobson A (1997). Population versus clinical view of case fatality from acute coronary heart disease: results from the WHO MONICA Project 1985–1990. Multinational MONItoring of Trends and Determinants in CArdiovascular Disease. Circulation.

[2] Buja LM (2005). Myocardial ischemia and reperfusion injury. Cardiovasc Pathol.

[3] Yellon DM, Hausenloy DJ (2007). Myocardial reperfusion injury. N Engl J Med.

[4] Fliss H, Gattinger D (1996). Apoptosis in ischemic and reperfused rat myocardium. Circ Res.

[5] Saraste A, Pulkki K, Kallajoki M (1997). Apoptosis in human acute myocardial infarction. Circulation.

[6] Haunstetter A, Izumo S (1998). Apoptosis: basic mechanisms and implications for cardiovascular disease. Circ Res.

[7] Xu H, Yu X, Qu S, Chen Y, Wang Z, Sui D (2014). Protective effect of *Panax quinquefolium* 20(S)-protopanaxadiol saponins, isolated from *Panax quinquefolium*, on permanent focal cerebral ischemic injury in rats. Exp Ther Med.

[8] Lin G, Yu X, Wang J, Qu S, Sui D (2013). Beneficial effects of 20(S)-protopanaxadiol on antitumor activity and toxicity of cyclophosphamide in tumor-bearing mice. Exp Ther Med.

[9] Wang LC, Lee TF (2000). Effect of ginseng saponins on cold tolerance in young and elderly rats. Planta Med.

[10] Shin JY, Song JY, Yun YS (2002). Immunostimulating effects of acidic polysaccharides extract of *Panax ginseng* on macrophage function. Immunopharmacol Immunotoxicol.

[11] Nishijo H, Uwano T, Zhong YM, Ono T (2004). Proof of the mysterious efficacy of ginseng: basic and clinical trials: effects of red ginseng on learning and memory deficits in an animal model of amnesia. J Pharmacol Sci.

[12] Li G, Wang Z, Sun Y, Liu K, Wang Z (2006). Ginsenoside 20(S)-protopanaxadiol inhibits the proliferation and invasion of human fibrosarcoma HT1080 cells. Basic Clin Pharmacol Toxicol.

[13] Wang T, Yu X, Qu S, Xu H, Han B, Sui D (2010). Effect of ginsenoside Rb3 on myocardial injury and heart function impairment induced by isoproterenol in rats. Eur J Pharmacol.

[14] Wang T, Yu XF, Qu SC, Xu HL, Sui DY (2010). Ginsenoside Rb3 inhibits angiotensin II-induced vascular smooth muscle cells proliferation. Basic Clin Pharmacol Toxicol.

[15] Shi Y, Han B, Yu X, Qu S, Sui D (2011). Ginsenoside Rb3 ameliorates myocardial ischemia-reperfusion injury in rats. Pharm Biol.

[16] Zhao ZQ, Corvera JS, Halkos ME (2003). Inhibition of myocardial injury by ischemic postconditioning during reperfusion: comparison with ischemic preconditioning. Am J Physiol Heart Circ Physiol.

[17] Nachlas MM, Shnitka TK (1963). Macroscopic identification of early myocardial infarcts by alterations in dehydrogenase activity. Am J Pathol.

[18] Gupta S (2001). Molecular steps of death receptor and mitochondrial pathways of apoptosis. Life Sci.

[19] Werns SW, Lucchesi BR (1989). Myocardial ischemia and reperfusion: the role of oxygen radicals in tissue injury. Cardiovasc Drugs Ther.

[20] Bolli R (1988). Oxygen-derived free radicals and postischemic myocardial dysfunction (‘stunned myocardium’). J Am Coll Cardiol.

[21] Ren G, Dewald O, Frangogiannis NG (2003). Inflammatory mechanisms in myocardial infarction. Curr Drug Targets Inflamm Allergy.

[22] Frangogiannis NG, Smith CW, Entman ML (2002). The inflammatory response in myocardial infarction. Cardiovasc Res.

[23] Antonsson B (2001). Bax and other pro-apoptotic Bcl-2 family ‘killer-proteins’ and their victim the mitochondrion. Cell Tissue Res.

[24] Budihardjo I, Oliver H, Lutter M, Luo X, Wang X (1999). Biochemical pathways of caspase activation during apoptosis. Annu Rev Cell Dev Biol.

